# Change in pulmonary diffusion capacity in a general population sample over 9 years

**DOI:** 10.3402/ecrj.v3.31265

**Published:** 2016-09-02

**Authors:** Michael L. Storebø, Tomas M. L. Eagan, Geir E. Eide, Amund Gulsvik, Einar Thorsen, Per S. Bakke

**Affiliations:** 1 Department of Thoracic Medicine, Haukeland University Hospital, Bergen, Norway; 2 Department of Clinical Science, University of Bergen, Bergen, Norway; 3 Centre for Clinical Research, Haukeland University Hospital, Bergen, Norway; 4 Life Style Epidemiology Research Group, Department of Global Public Health and Primary Care, University of Bergen, Bergen, Norway

**Keywords:** diffusion capacity for carbon monoxide, longitudinal change, occupational exposure, socioeconomic status, smoking

## Abstract

**Rationale:**

Data on the change in diffusion capacity of the lung for carbon monoxide (DL_CO_) over time are limited. We aimed to examine change in DL_CO_ (ΔDL_CO_) over a 9-year period and its predictors.

**Methods:**

A Norwegian community sample comprising 1,152 subjects aged 18–73 years was examined in 1987 and 1988. Of the 1,109 subjects still alive, 830 (75%) were re-examined in 1996/97. DL_CO_ was measured with the single breath-holding technique. Covariables recorded at baseline included sex, age, height, weight, smoking status, pack years, occupational exposure, educational level, and spirometry. Generalized estimating equations analyses were performed to examine relations between ΔDL_CO_ and the covariables.

**Results:**

At baseline, mean [standard deviation (SD)] DL_CO_ was 10.8 (2.4) and 7.8 (1.6) mmol·min^−1^·kPa^−1^ in men and women, respectively. Mean (SD) ΔDL_CO_ was −0.24 (1.31) mmol·min^−1^·kPa^−1^. ΔDL_CO_ was negatively related to baseline age, DL_CO_, current smoking, and pack years, and positively related to forced expiratory volume in 1 second (FEV_1_) and weight. Sex, occupational exposure, and educational level were not related to ΔDL_CO_.

**Conclusions:**

In a community sample, more rapid decline in DL_CO_ during 9 years of observation time was related to higher age, baseline current smoking, more pack years, larger weight, and lower FEV_1_.

Diffusing capacity of the lung for carbon monoxide (DL_CO_) is the most widely used non-invasive test of pulmonary gas transfer (1). The test has been used in both clinical and epidemiological settings and in surveys of occupational groups (2–8). Several cross-sectional community studies have presented predictors for DL_CO_ (9–17), and commonly used reference values are based on sex, age, and height. In some cross-sectional studies, smoking has been found to be associated with impaired DL_CO_, while body mass and socioeconomic status (SES) have been shown to be related to DL_CO_ in some studies (14, 17). Only two community studies have been longitudinal in design, which is preferable to cross-sectional studies when studying change related to ageing (18, 19).

The two longitudinal studies were an 8-year follow-up study from Tucson, Arizona (18), including 543 subjects, and an 8-year follow-up study from Pisa, Italy, including 928 subjects (19). Both studies found that the decline in DL_CO_ during the follow-up period increased with increasing age, while no relationship to smoking was noted. The latter is somewhat surprising as smoking is the major cause of emphysema, which is associated with impaired DL_CO_ (20). A small cohort study of 84 subjects, followed for 22 years, has observed smoking to be a predictor for rapid decline of DL_CO_ (21, 22). The representativity of this cohort to the population at large is uncertain.

The purpose of this study was to explore predictors for the longitudinal change in DL_CO_ in a community sample examined twice 9 years apart. According to previous findings in cross-sectional studies of this population sample (17, 23–26), we hypothesized that smoking habits, occupational airborne exposure, and SES were predictors of change in DL_CO_.

## Methods

### Study population

Details of the sampling and characterization of the study population have been given elsewhere (27, 28). Briefly, a stratified sample (*n*=1,512) from the general population in Hordaland, Norway, aged 18–73 years was invited to a clinical and respiratory physiological examination in 1987/88. Altogether 1,275 (84%) attended. DL_CO_ measurements were obtained from 1,152 (90%) of the 1,275 attendees.

All attendees from visit 1 were invited to a follow-up (visit 2) in 1996/97. From the 1,152 subjects with DL_CO_ measurements at visit 1, 881 (76%) attended visit 2. Of those lost to follow-up, 43 were dead, 81 no longer lived in the study area, 63 did not wish to participate further, and 23 could not attend because of serious illness. We were not able to establish contact with 61 of the visit 1 attendees. We obtained DL_CO_ measurements from 830 (94%) of the visit 2 attendees.

### Questionnaires

At visit 1, data on smoking habits, educational level, and occupational airborne exposure were obtained through self-reported questionnaires (23, 29). Smoking habit was categorized into never smoking, ex-smoking, and current smoking. Pack years was calculated as average number of cigarettes smoked per day, divided by twenty and multiplied by total number of years of being a smoker. SES was assessed in terms of educational level which was categorized into primary school, secondary school, and higher education (17).

Occupational airborne exposure was based on the following data: self-reported past or present occupational exposure to dust or gas (24) and self-reported exposure to specific agents and work processes (asbestos, quartz, wood dust, welding, and soldering) (27).

### Clinical examination and pulmonary function testing

Clinical examination included measurements of height and weight. Blood samples were analyzed for hemoglobin (Hb) concentration and fraction of carboxyhemoglobin (HbCO). Pulmonary function testing (PFT), including DL_CO_, and forced spirometry were performed in accordance with current guidelines at the time of examination (1, 30–32).

PFT at both visit 1 and visit 2 was performed using a SensorMedics Gould 2100 automated system (SensorMedics BV, Bilthoven, the Netherlands). The same instrument was used at both visits, with the same calibration procedure and biological control throughout the observation period by regular measurements of the technicians operating the instrument. Details of the standardization of measurements, calibration processes, and the results of repeated measurements in the biological controls are given in the Supplementary file. At both visits, DL_CO_, the alveolar volume (V_A_), and the ratio of DL_CO_ to V_A_ (K_CO_) were measured using the single breath-holding method, with a breath holding time of 10 seconds, a washout volume of 0.75 L, and a sample volume of 0.75 L. V_A_ was measured by helium dilution. The test gas was delivered and certified by Norsk Hydro A/S (Rjukan, Norway). The concentration of carbon monoxide was requested to be within 0.270 and 0.330% with an accuracy of 1%. The concentration of helium was requested to be within 9 and 11% with an accuracy of 1%. The mean of two measurements, with no more than 10% variability, is reported. The ATS/ERS guidelines require the DL_CO_ measurement to be performed after the subject had achieved an inspiratory vital capacity (IVC) of at least 85% of his or her forced vital capacity (FVC) (27). Only 531 subjects (64%) met this criterion on both visits, while 750 subjects (90%) achieved an IVC/FVC ratio of at least 0.7. Excluding the subjects with an IVC/FVC ratio of less than 0.85 did not alter the study results overtly as compared to including them in the analyses (Tables E1 and E2). Hence, the data are presented including all subjects with an IVC/FVC ratio>0.7. Predicted values for DL_CO_ were calculated using the formula estimated by Cotes et al. (1). It was decided not to use Norwegian predicted values, as they are based on the population sample also used in this study.

Spirometry was performed as an inhalation from functional residual capacity to total lung capacity, followed by a maximal forced expiration to residual volume. For forced expiratory volume in 1 second (FEV_1_) and FVC, the highest value from three technically acceptable measurements, with variability between the two highest values within 300 mL, is reported. All subjects were shown how to perform the maneuvers before testing, using standardized instructions, for both forced spirometry and measurement of DL_CO_. Subjects were seated and wearing a nose-clip during all efforts. Reference values calculated from healthy Norwegian subjects were used for FEV_1_ (26).

### Statistical methods

Descriptive statistics are presented using the mean and standard deviation (SD) for continuous variables and frequency and percentage for categorical variables. Comparisons of the study population and those lost to follow-up were performed using the independent samples t-test and the exact chi-squared test. Comparisons of means from baseline and follow-up were performed using paired samples t-test, testing for cohort effect was carried out using independent samples t-test, and modeling change in DL_CO_ as a function of age was performed using curve estimation. Testing for normal distribution was performed using the Kolmogorov-Smirnov and the Shapiro-Wilk tests.

DL_CO_ at first and follow-up survey 9 years later was analyzed in a multiple linear regression model and estimated with generalized estimating equations (GEE) to account for correlation between the two measures of DL_CO_ in the same subject at the two surveys. In this model, time was given the values 0 and 9 (years), all other continuous explanatory variables were centered around their means, all categorical variables were represented by dummy variables, and all interactions between the explanatory variables (categorical and continuous) were included. From such a model, the estimated regression coefficients for the interactions give direct estimates of the average yearly change in DL_CO_ from the first to the last visit (ΔDL_CO_) at the zero level for all explanatory variables (for continuous variables this is the mean value; for categorical variables it is the reference category), and for a value of 1 unit increase from 0 in each variable all others were fixed at 0. For the GEE estimation, an exchangeable correlation structure was assumed.

Models with adjustments for change in Hb and HbCO were also made. Finally, we decided a priori to test the following interactions: age versus sex, age versus smoking habits, and sex versus smoking habits. A significance level of 5% was used for all analyses.

SPSS version 20 (IBM Corporation, New York, USA) was used for all analyses except for the GEE estimation for which Stata version 12 (StataCorp, College Station, Texas, USA) was applied.

## Results

### Study population description

The characteristics of those examined at baseline and at follow-up and those lost to follow-up are outlined in Table 1. Almost half of the sample was ever-smokers, and approximately one quarter of the subjects was current smokers. Those who were lost to follow-up were significantly older and had significantly lower lung function than those who remained in the study.

**Table 1:** Descriptive statistics for characteristics at baseline and follow-up of the stratified sample from the general population in Hordaland County, Norway, aged 18–73 years in 1987/88 with follow-up 9 years later

	Baseline	Follow-up	Lost to follow-up
	
Variable	*n*=1,152	*n*=830	*n*=322
Sex (male), n (%)	590 (51.2)	436 (52.5)	154 (47.8)
Age (years), mean (SD)	41.6 (16.0)	49.8 (14.4)	44.4 (19.3)
Height (cm), mean (SD)	171.8 (9.3)	172.1 (9.4)	170.1 (9.3)
Weight (kg), mean (SD)	71.4 (12.8)	75.9 (13.9)	69.7 (12.1)
Smoking habits, n (%)			
Daily smokers	310 (26.9)	233 (24.7)	77 (23.9)
Ex-smokers	207 (18.0)	149 (21.8)	58 (18.0)
Never smokers	635 (55.1)	448 (53.5)	187 (58.1)
Pack years smoked,^a^ mean (SD)	12.7 (11.1)	16.1 (12.3)	13.7 (14.1)
Occupational exposure, n (%)	337 (29.3)	259 (31.2)	78 (24.2)
Education level, n (%)			
Primary school	213 (18.5)	133 (16.0)	80 (24.8)
Secondary school	714 (62.0)	532 (64.1)	182 (56.5)
Higher education	225 (19.5)	165 (19.9)	60 (18.6)
FEV_1_ (L), mean (SD)	3.60 (1.02)	3.28 (0.96)	3.33 (1.12)
FEV_1_ percent predicted, mean (SD)	95 (14)	92 (15)	92 (16)
DL_CO_ (mmol·min^−1^·kPa^−1^), mean (SD)	9.37 (2.53)	9.35 (2.61)	8.81 (2.67)
DL_CO_ percent predicted, mean (SD)	94 (15)	98 (18)	91 (17)

SD, standard deviation; FEV_1_, forced expiratory volume in 1 second; DL_CO_, diffusing capacity of the lung for carbon monoxide.

a Non-smokers excluded.

Analyses were performed to discover a cohort effect, if present, by comparing baseline FEV_1_ and DL_CO_ values of those aged 40–44 years at baseline with the corresponding follow-up values of those aged 40–44 years at visit 2. Analyses were performed independently for men and women to adjust for difference in the ratio between the sexes in these sub-samples. There were no statistically significant differences in mean values of FEV_1_ and DL_CO_.

### Baseline DL_CO_

Mean DL_CO_ at baseline for the entire cohort (*n*=1,152) was 9.37 mmol·min^−1^·kPa^−1^ (SD: 2.53). Using multiple linear regression, we found that female sex, higher age, current smoking, ex-smoking, and increased pack years were associated with lower DL_CO_. Higher body height, larger weight, and higher FEV_1_ were significantly associated with higher baseline DL_CO_, as was higher education compared to secondary school. Occupational airborne exposure was not associated with baseline DL_CO_ regardless of whether the exposure characterization was based on self-reported dust or gas or self-reported exposure to specific airborne agents (Table 2, and Tables E3 and E4).

**Table 2:** Descriptive statistics for baseline DL_CO_ in 1987/88 and average change per year during a 9-year follow-up, ΔDL_CO_, for 830 subjects from Hordaland County, Norway, according to baseline characteristics

Characteristics at baseline	Baseline DL_CO_ (mmol·min^−1^·kPa^−1^), mean (SD)	ΔDL_CO_ (mmol·min^−1^·kPa^−1^·year^−1^), mean (SD)
Sex		
Male	10.85 (2.38)	−0.039 (0.161)
Female	7.83 (1.57)	−0.010 (0.114)
Age in years		
Up to 19	10.60 (2.39)	0.003 (0.158)
20–29	10.88 (2.49)	−0.021 (0.150)
30–39	10.00 (2.20)	0.001 (0.129)
40–49	9.45 (2.10)	−0.037 (0.163)
50–59	8.23 (2.01)	−0.032 (0.134)
60–69	7.54 (1.69)	−0.072 (0.103)
70–79	6.02 (1.46)	−0.050 (0.122)
Height in cm		
159 and below	6.55 (1.27)	−0.023 (0.118)
160–169	7.90 (1.61)	−0.018 (0.103)
170–179	9.93 (1.97)	−0.030 (0.142)
180–189	11.62 (2.31)	−0.034 (0.192)
190 and above	12.84 (2.16)	−0.005 (0.154)
Weight in kg		
− 49	6.08 (1.80)	0.001 (0.114)
50–59	7.76 (1.64)	−0.016 (0.111)
60–69	8.83 (2.24)	−0.026 (0.120)
70–79	10.06 (2.54)	−0.041 (0.156)
80–89	10.48 (2.41)	−0.001 (0.150)
90–99	10.61 (2.44)	−0.034 (0.207)
100	10.78 (2.89)	−0.049 (0.118)
Smoking habits		
Never smoker	9.62 (2.62)	−0.012 (0.144)
Ex-smoker	9.20 (2.31)	−0.037 (0.119)
Daily smoker	8.99 (2.43)	−0.044 (0.148)
Pack years smoked		
0	9.62 (2.62)	−0.012 (0.144)
1–20	9.23 (2.40)	−0.031 (0.136)
21–40	8.75 (2.19)	−0.080 (0.137)
> 40	6.79 (1.92)	−0.094 (0.125)
Occupational exposure		
No	9.08 (2.32)	−0.019 (0.138)
Yes	10.12 (2.53)	−0.029 (0.152)
Education level		
Primary school	8.15 (2.22)	−0.041 (0.131)
Secondary school	9.43 (2.44)	−0.023 (0.144)
Higher education	10.37 (2.62)	−0.020 (0.143)
FEV_1_ quartiles		
2.89 L and below	6.87 (1.51)	−0.031 (0.109)
2.90–3.55 L	8.56 (1.27)	−0.030 (0.125)
3.56–4.36 L	9.95 (1.66)	−0.014 (0.145)
4.37 and above	12.20 (1.95)	−0.029 (0.174)

DL_CO,_ diffusing capacity of the lung for carbon monoxide; FEV_1,_ forced expiratory volume in 1 second; SD, standard deviation.

### Change in DL_CO_

Mean DL_CO_ at follow-up (*n*=830) was 9.35 mmol·min^−1^·kPa^−1^ (SD: 2.61). Baseline DL_CO_ for the same 830 participants was 9.59 mmol·min^−1^·kPa^−1^ (SD: 2.44). Mean ΔDL_CO_ between baseline and follow-up for those who attended both visits was −0.24 mmol·min^−1^·kPa^−1^ (95% CI: −0.33 to −0.15).

Mean change in DL_CO_ percent of predicted values for those subjects who attended both visits was 3.0% (95% CI: 2.3 to 4.1). Mean change in FEV_1_ percent of predicted values for the same subjects was −3.0% (95% CI −3.9 to −2.7).

**Fig. 1:**
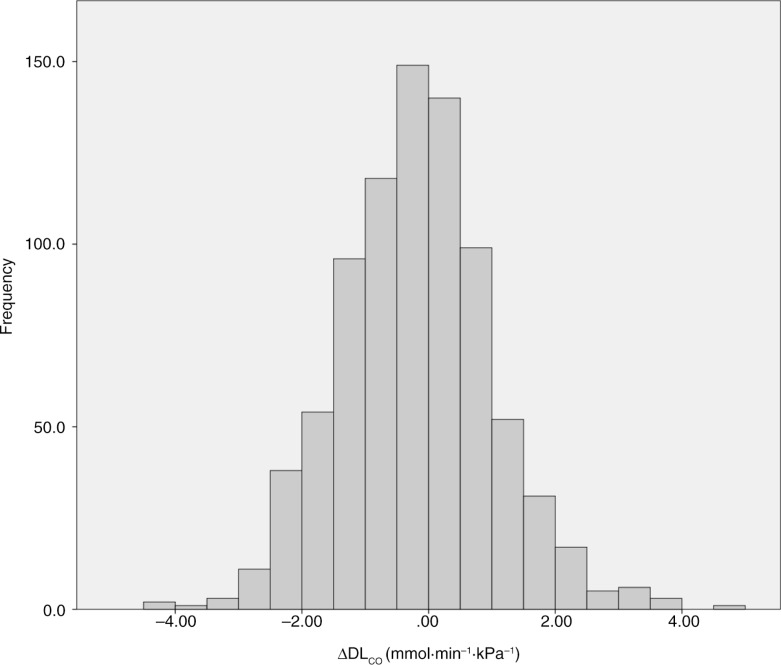
The distribution of change in DL_CO_ during a 9-year follow-up from 1987/88 in 830 subjects from Hordaland County, Norway.

ΔDL_CO_ had a normal distribution, tested by both the Kolmogorov-Smirnov and the Shapiro-Wilk tests, with a large variation (Fig. 1). Approximately 40% had a decline of more than twice the average, while 5% had no change (0±0.10 mmol·min^−1^·kPa^−1^), and 38% had an increase (>0.10 mmol·min^−1^·kPa^−1^).

Univariate associations using GEE, adjusting only for baseline DL_CO_ and change in Hb concentration and HbCO, were found for age, height, baseline FEV_1_, smoking habits, and pack years.

The multivariate analysis, including baseline DL_CO_, sex, age, baseline height, baseline weight, baseline FEV_1_, baseline smoking habits, pack years smoked before baseline, occupational exposure, and educational level, showed that higher baseline DL_CO_ and age were associated with a more rapid decline in DL_CO_. Current smokers had a more rapid decline than never smokers, and increased pack years was associated with more rapid decline as well. Higher body height and weight, and higher FEV_1_ were associated with a lower rate of decline in DL_CO_. All the associations above persisted after adjusting for change in Hb and HbCO. Sex, occupational exposure to gas or dust, and level of education were not significantly associated with ΔDL_CO_ in the multivariate analyses (Table 3).

**Table 3:** Adjusted yearly change in DL_CO_ estimated by generalized estimating equations (GEE) of the stratified sample from the general population in Hordaland County, Norway, aged 18–73 years in 1987/88 with follow-up 9 years later

Characteristic at baseline	Estimate	*p*
DL_CO_ at baseline		
At DL_CO_ 9.6	−0.0293	
Per 1 unit increase	−0.0325	<0.0001
Age at baseline		
At age 45 years	−0.0293	
Per 10 years increase	−0.0243	<0.0001
Sex		
Men	−0.0293	
Women	−0.0162	0.410
Height at baseline		
At 170 cm	−0.0293	
Per 10 cm increase (at baseline)	0.0240	0.013
Weight at baseline		
At 70 kg	−0.0293	
Per 1 kg increase	0.0011	0.020
Smoking at baseline		0.001
Never	−0.0293	
Ex	−0.0238	0.700
Current	−0.0738	0.002
Pack years smoked before baseline		
At 6 pack years	−0.0293	
Per 10 pack years increase	−0.0196	0.003
Occupational exposure		
No	−0.0293	
Yes	0.0146	0.177
Educational level		0.310
Primary school	−0.0293	
Secondary school	−0.0443	0.270
Higher education	−0.0304	0.947
FEV_1_ at baseline		
At FEV_1_ 3.6 L	−0.0293	
Per 1 L increase	0.0235	0.013

DL_CO,_ diffusing capacity of the lung for carbon monoxide in mmol·min^−1^·kpa^−1^; FEV_1_, forced expiratory volume in 1 second.

We found no interactions between age and sex, age and smoking habits, or sex and smoking habits on change in DL_CO_.

Mean alveolar volume (V_A_) was 6.49 L (SD: 1.30) at baseline and 6.29 L (SD: 1.38) at follow-up. There was a significant reduction in V_A_ during the observation period. In a multivariate analysis, higher baseline V_A_ and female sex were significant predictors of a more rapid decline in V_A_ (Table E5).

Mean carbon monoxide diffusion coefficient (K_CO_) at baseline was 1.48 mmol·min^−1^·kPa^−1^·L^−1^ (SD: 0.25) and 1.49 mmol·min^−1^·kPa^−1^·L^−1^ (SD: 0.32) at follow-up. When analyzing the values from only the participants who met the requirement of an IVC/FVC ratio of 0.85 or above, the corresponding means were 1.45 mmol·min^−1^·kPa^−1^·L^−1^ (SD: 0.24) and 1.46 mmol·min^−1^·kPa^−1^·L^−1^ (SD: 0.28), respectively. When analyzed in a multivariate model, we found that higher baseline K_CO_, male sex, higher age, lower baseline body weight, current smoking, higher number of pack years smoked, and lower level of education were significant predictors of a more rapid decline in K_CO_ (Table E6).

## Discussion

In this 9-year follow-up study of a general population sample, we observed that the rate of decline in gas diffusion capacity was highly variable. Mean change in DL_CO_ was −0.025 mmol·min^−1^·kPa^−1^·year^−1^. Current smoking was the strongest predictor for decline in DL_CO_. In addition, older age, higher cumulative smoking consumption in terms of pack years, lower level of FEV_1_, lower body weight, and shorter body height were independent predictors of increased DL_CO_ loss. Sex, educational level, and occupational airborne exposure did not independently influence change in DL_CO_.

This is the first community study to show that current smoking status and previous smoking consumption in terms of pack years predict loss of DL_CO_. The study is also the first to examine the effect of educational level and occupational airborne exposure on change in gas diffusion capacity. Our study confirms the findings of others (18, 19) that the decline in DL_CO_ becomes more rapid with higher age.

The magnitude of the decline in DL_CO_ observed in our study is comparable to that found by Viegi et al. (19), while comparison to the decline found by Sherrill et al. (18) is more complicated because of differences in how the results are reported. Standard error of the mean of DL_CO_ seems to be comparable between all three studies.

Current smoking was related to a reduced baseline DL_CO_ and a larger subsequent decline in DL_CO_ in the multivariate analyses. Adjusting for HbCO did not change this association. Hence, current smoking has an effect on level and decline of DL_CO_ beyond that of previous exposure and that of HbCO. Smokers more often develop anemia that may impair gas diffusion (33). However, when change in Hb was added to the equation, the relationship between smoking and DL_CO_ persisted. The study was not designed to investigate mechanisms by which tobacco smoke could alter the rate of change in DL_CO._

Cumulative smoking exposure in terms of pack years was also an independent predictor of future decline in DL_CO_ (Table 3). There may be several explanations for this finding. First, smoking exposure may cause airflow limitation and air trapping that lead to impaired gas diffusion capacity. However, the effect of pack years on DL_CO_ decline persisted after taking baseline FEV_1_ into account (Table 3). Second, we have recently shown in another data set that level of emphysema is related to DL_CO_ after adjusting for FEV_1_ (34). Hence, increased smoking consumption may cause decline in DL_CO_ because of more emphysema.

Neither the Italian nor the American community study observed that current smoking or smoking consumption was related to decline in DL_CO_ (18, 19). The follow-up rate in the Italian study was lower than that in the current study, and smokers tend to drop out more often than non-smokers in longitudinal surveys (35). The American study comprised only about half the number of subjects of our study and they had no subjects above the age of 59 years at baseline (18).

In line with others (18, 19), we observed that the DL_CO_ decline becomes more rapid with increasing age. The best fit of the model was for age squared, adding further support to our finding that the decline accelerated with increasing age. In the multivariate analysis, this acceleration in the decline with increasing age was found to be independent of smoking, lung function, body height and weight, as well as occupational exposure and SES. Potential explanations might be age-related reduced alveolar ventilation, increased level of emphysema, increased pulmonary blood pressure, and impaired cardiac function (36).

When comparing DL_CO_ with available European predicted values, we observed an increase in the percent predicted value while there was a decrease in the absolute value. These predicted values were based on a compilation of European cross-sectional studies, and the age coefficient may be overestimated because of a cohort effect and less precise characterization of the subjects with respect to symptoms, previous smoking, and occupational exposure. As for FEV_1_, the annual change in longitudinal studies is less than the estimated annual change from cross-sectional surveys.

The difference between cross-sectional and longitudinal estimates of annual change may also be influenced by regression to the mean. We included baseline DL_CO_ in the model which will partially account for that phenomenon.

We did not observe that occupational airborne exposure influenced level of DL_CO_ or decline of DL_CO_ in this general population sample. This may imply that there is no impact of occupational exposure on gas diffusion capacity in a community setting, or that we have not been able to show it. Regarding the latter possibility, the exposure characterization applied in the present study has been used to show a relationship between lung function in terms of spirometry (27, 37), diagnosis of asthma and chronic obstructive pulmonary disease (27, 38), as well as the prevalence and incidence of respiratory symptoms (24, 38). The exposure data have a high specificity, but a lower sensitivity (29). Those stating exposure have in general been exposed to a higher degree than those falsely stating no exposure (29). Hence, we think that our study indicates that the level of occupational exposure in a general population sample is not high enough to cause impaired level of DL_CO_ and more rapid decline in DL_CO_.

We have previously shown in cross-sectional analyses in this population that lower SES in terms of educational achievement is independently related to reduced level of DL_CO_ (17). However, we did not observe that SES predicted subsequent change in DL_CO_ after adjusting for the other covariates. As people tend to stay in the socioeconomic class into which they are born, the effect of SES on DL_CO_ may have been evident at an early stage in life after which the subsequent decline in DL_CO_ is independent of SES. However, it should be noted that low as compared to high SES was an independent predictor of rapid decline in K_CO_ (Table E6).

### Strengths and limitations of the study

This study is based on a community survey with high response rates both at baseline and follow-up. The study sample is representative of the population at large with respect to sex, age, and smoking (25, 35). Except for the requirement of an IVC/FVC ratio above 0.85, the participants included in the analyses met the ATS-criteria for a satisfactory DL_CO_ test (28). The same equipment for measuring DL_CO_ was used at baseline and follow-up with the same technicians. The effect of smoking on change in DL_CO_ was adjusted for by change in HbCO, and finally validated questions on occupational exposure were used.

There are also some limitations to the study. First, we had only two points of observations, rendering the study susceptible to regression towards the mean. On the other hand, we adjusted for baseline level of DL_CO_, which should at least partly take this bias into account. Second, we did not have data on menstrual cycle for female participants, and are therefore not able to adjust for the effects of the menstrual cycle on DL_CO_ (39–41).

In conclusion, we have observed that in the population at large both current smoking and cumulative smoking exposure, reduced FEV_1_, and increasing age predict more rapid decline in gas diffusion capacity, while occupational exposure and SES do not. This knowledge may help physicians in their interpretation of DL_CO_ measurements.

## Supplementary Material

Change in pulmonary diffusion capacity in a general population sample over 9 years

Change in pulmonary diffusion capacity in a general population sample over 9 years
