# Supplementary material for: Change in pulmonary diffusion capacity in a general population sample over 9 years
Source: Eur Clin Respir J. 2016 Sep 2;3:10.3402/ecrj.v3.31265. doi: 10.3402/ecrj.v3.31265 (PMC5013260; doi:10.3402/ecrj.v3.31265)
Supplement: Change in pulmonary diffusion capacity in a general population sample over 9 years [file ECRJ-3-31265-s001.docx]

**Online supplement: Calibration and standardisation procedures**

A manual with instructions for all measurements an calibration procedures was made before data collection begun. The ATS guidelines by Ferris (1) were used extensively in the development of the study manual. For standardisation of spirometry, the guidelines from European Community for Coal and Steel was used (2).

Body height was measured without shoes and rounded to the closest centimeter.

Body weight was measured without shoes and clothes on the upper body. Trousers were left on, but pockets emtpied. Weight was rounded to the closest kilogram.

A mercury barometer and mercury thermometer was used to register temperature and atmospheric pressure before pulmonary function testing for every subject.

Flow calibration was performed every morning, using both an automated and manual procedure. The spirometer used two hot wire anemometers: One for inspiration and one for expiration. Calibration syringes were calibrated by filling them with water, and weighing the contents. For automated calibration, a motorized syringe, with the plunger connected to an eccentric sheave, pumped 2.15L of air through the system four times. For manual calibration, a Gould Model M-20 calibration syringe of 3.00L was used to pump air through the system six times. A measurement variation of up to +/- 3% from the syringe volumes was accepted.

Gas analyzer calibration was performed before each examination, with a certified test gas from Norsk Hydro, containing 0.300% carbon monoxide, 10% helium, 21% oxygen and a balance of nitrogen. A variation of +/- 0.1% from the known test gas concentrations was accepted.

In addition, pulmonary function testing was performed on biological controls every morning to detect any errors not detected by the calibration processes. Data from on of the controls are summarized in figure S1.

**References**

1. Ferris BG. Epidemiology Standardization Project (American Thoracic Society). Am Rev Respir Dis. 1978;118(6 Pt 2):1-120.

2. Quanjer P DA, Van Zoramen B. Standardised Lung Function Testing. Report of the working party for the European Community for Coal and Steel. Bull Eur Physiopathol Respir. 1983;19 Suppl 5:1-95.

**Figure S1** DL_CO_ measurement results from a biological control


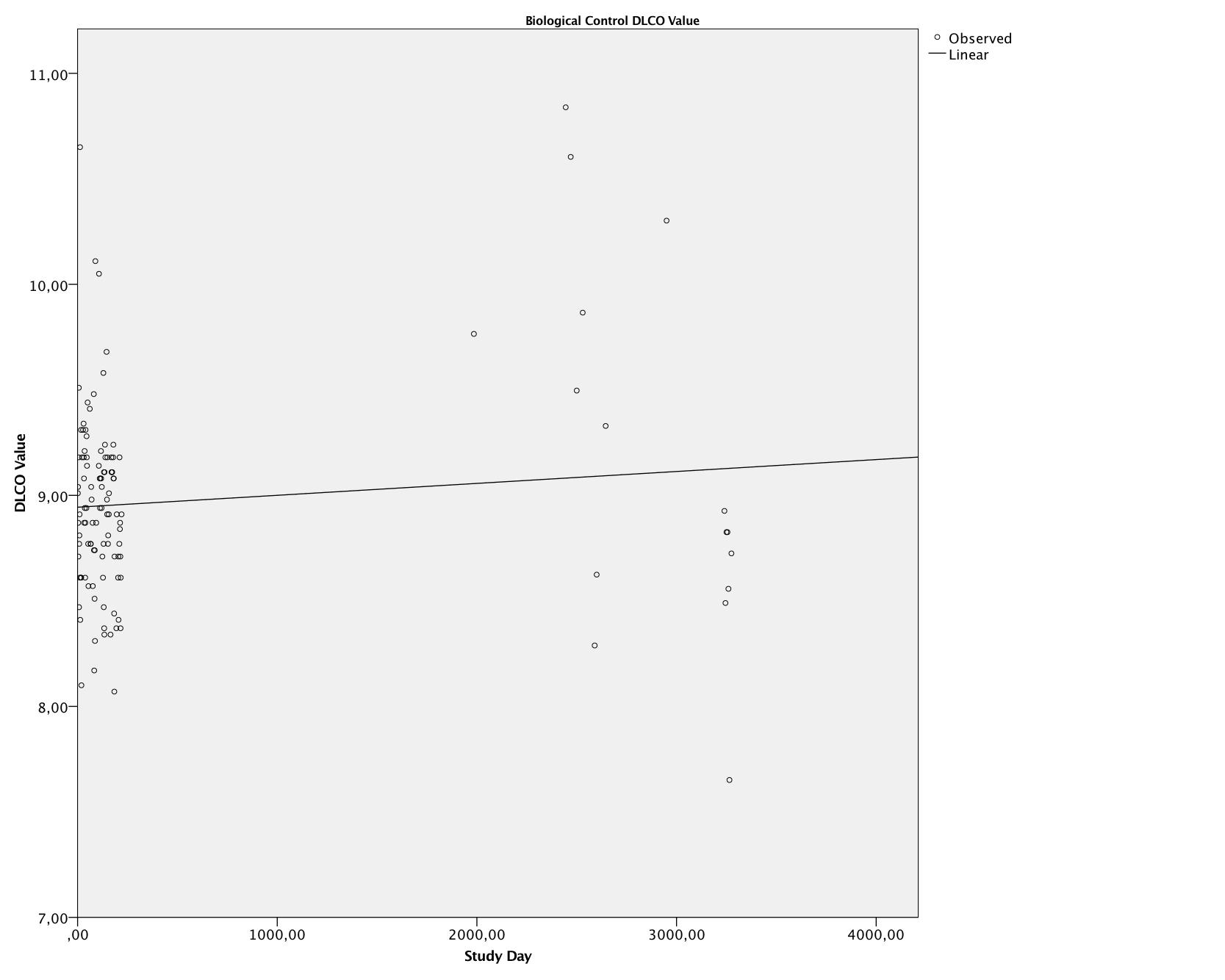


**Figure S2** DL_CO_ measurement results from a biological control

**
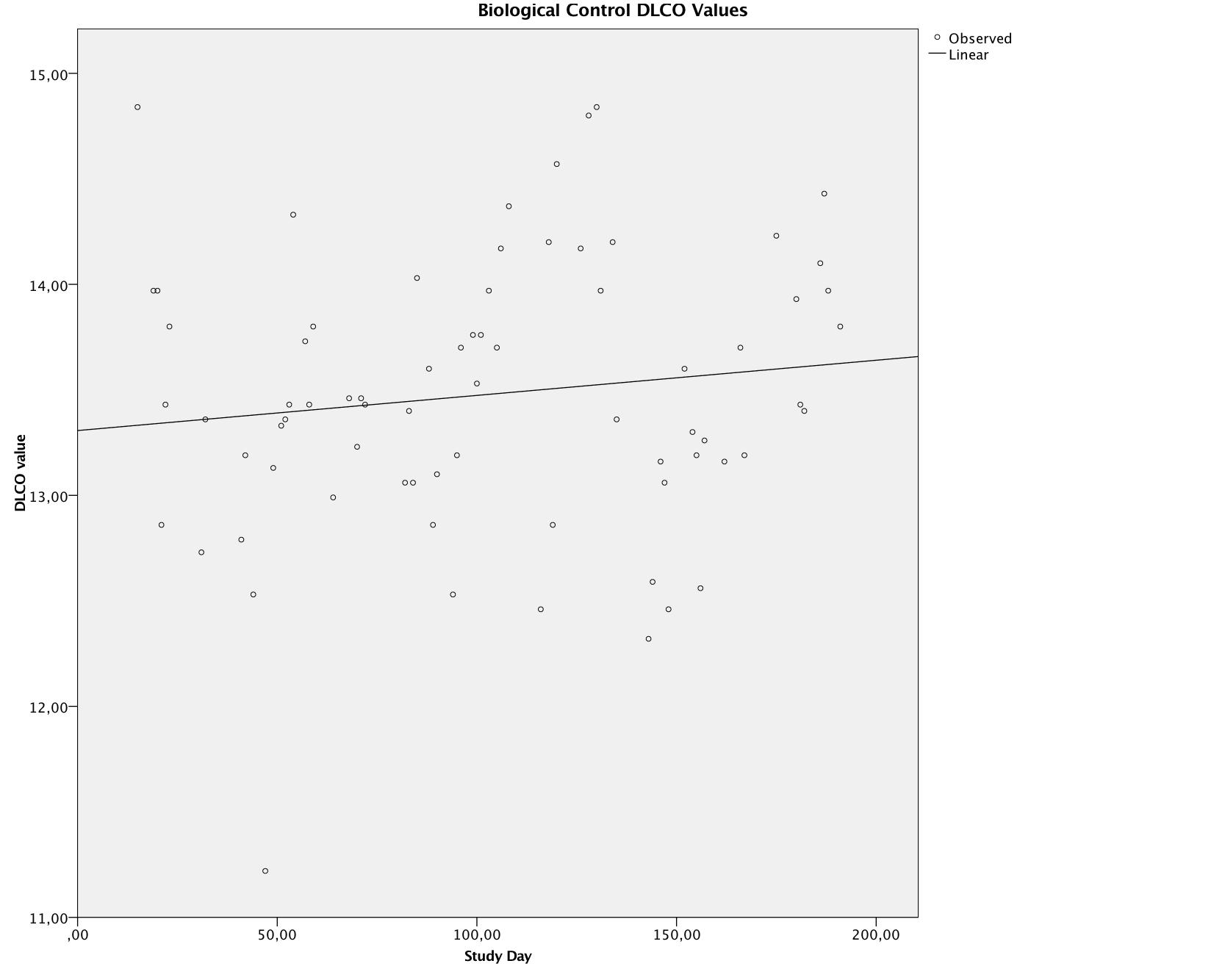
**
