# Supplementary material for: Change in pulmonary diffusion capacity in a general population sample over 9 years
Source: Eur Clin Respir J. 2016 Sep 2;3:10.3402/ecrj.v3.31265. doi: 10.3402/ecrj.v3.31265 (PMC5013260; doi:10.3402/ecrj.v3.31265)
Supplement: Change in pulmonary diffusion capacity in a general population sample over 9 years [file ECRJ-3-31265-s002.docx]

**Online supplement: Calculating predicted change in DL_CO_**

Predicted DL_CO_ can be calculated using the values from table 3 using the following formula:

*Predicted ΔDL_CO_ = (-0.0293+((DL_CO_-9.6)·(-0.0325)))+(-0.0293+(((Age-45)·10^-1^)·(-0.0243)))+(-0.0293 for men | -0.0162 for women)+(-0.0293+(((Height-170)·10^-1^)·0.0240))+(-0.0293+((Weight-70)·0.0011))+(-0.0293 for never-smokers | -0.0238 for ex-smokers | -0.0738 for current smokers)+(-0.0293+(((Pack years-6)·10^-1^)·(-0.0196)))+(-0.0293 for no occupational exposure | 0.0146 for occupational exposure)+(-0.0293 if primary school | -0.0443 if secondary school | -0.0304 if higher education)+(-0.0293+((FEV_1_-3.6)·0.0235)*

A sample calculation for a subject with the following characteristics can be found beneath:

- DL_CO_ at baseline: 8.6 mmol · min^-1^ · kPa^-1^
- Age at baseline: 59
- Sex: Woman
- Height: 165cm
- Weight at baseline: 78kg
- Ex-smoker
- Pack years at baseline: 34
- No occupational exposure
- Educational level: Secondary school
- FEV_1_ at baseline: 2.78 L

*(-0.0293+((8.6-9.6)·(-0.0325)))+(-0.0293+(((59-45)·10^-1^)·(-0.0243)))+(-0.0162)+(-0.0293+(((165-170)·10^-1^)·0.0240))+(-0.0293+((78-70)·0.0011))+(-0.0238)+(-0.0293+(((34 -6)·10^-1^)·(-0.0196)))+(-0.0293)+(-0.0443)+(-0.0293+((2.78-3.6)·0.0235) = -0.36823 mmol · min^-1^ · kPa^-1^*

Predicted change in V_A_ and K_CO_ can be calculated in the same manner using the values from tables E5 and E6 respectively.
